# A three-arm, multicenter, open-label randomized controlled trial of hydroxychloroquine and low-dose prednisone to treat recurrent pregnancy loss in women with undifferentiated connective tissue diseases: protocol for the Immunosuppressant regimens for LIving FEtuses (ILIFE) trial

**DOI:** 10.1186/s13063-020-04716-1

**Published:** 2020-09-09

**Authors:** Shaoying Yang, Ruoning Ni, Yikang Lu, Suli Wang, Feng Xie, Chunyan Zhang, Liangjing Lu

**Affiliations:** 1 Department of Rheumatology, Renji Hospital, Shanghai Jiao Tong University School of Medicine, 145 Middle Shandong Road, Shanghai, 200001 China; 2 Department of Internal Medicine, Saint Agnes Hospital, Baltimore, MD USA; 3 Department of Health Research Methods, Evidence and Impact (formerly Clinical Epidemiology and Biostatistics), McMaster University, Hamilton, Ontario Canada; 4 Centre for Health Economics and Policy Analysis, Faculty of Health Sciences, McMaster University, Hamilton, Ontario Canada

**Keywords:** Prednisone, Hydroxychloroquine, Recurrent pregnancy loss, Recurrent spontaneous abortion, Undifferentiated connective tissue disease, Randomized controlled trial

## Abstract

**Background:**

Undifferentiated connective tissue disease (UCTD) is known to induce adverse pregnancy outcomes and even recurrent spontaneous abortion (RSA) by placental vascular damage and inflammation activation. Anticoagulation can prevent pregnancy morbidities. However, it is unknown whether the addition of immune suppressants to anticoagulation can prevent spontaneous pregnancy loss in UCTD patients. The purpose of this study is to evaluate the efficacy of hydroxychloroquine (HCQ) and low-dose prednisone on recurrent pregnancy loss for women with UCTD.

**Methods:**

The Immunosuppressant for Living Fetuses (ILIFE) Trial is a three-arm, multicenter, open-label randomized controlled trial with the primary objective of comparing hydroxychloroquine combined with low-dose prednisone and anticoagulation with anticoagulation alone in treating UCTD women with recurrent spontaneous abortion. The third arm of using hydroxychloroquine combined with anticoagulant for secondary comparison. A total of 426 eligible patients will be randomly assigned to each of the three arms with a 1:1:1 allocation ratio. The primary outcome is the rate of live births. Secondary outcomes include adverse pregnancy outcomes and progression of UCTD.

**Discussion:**

This is the first multi-center, open-label, randomized controlled trial which evaluates the efficacy of immunosuppressant regimens on pregnancy outcomes and UCTD progression. It will provide evidence on whether the immunosuppressant ameliorates the pregnancy prognosis in UCTD patients with RSA and the progression into defined connective tissue disease.

**Trial registration:**

ClinicalTrials.gov NCT03671174. Registered on 14 September 2018.

## Background

Undifferentiated connective tissue disease (UCTD) is defined as at least one symptom or sign suggesting connective tissue diseases (CTDs) and with presence of at least one of auto-antibodies, while not fulfilling any classification criteria of a defined CTD [1, 2]. UCTD accounts for 20–52% of patients with CTDs and is characterized by varying symptom onset time and antibody profiles. UCTD is more common in women, with a male-to-female ratio ranging from 1:15 to 1:17, predominantly at the reproductive age with a prevalence of 2.5% in pregnant women [2–5]. UCTD may evolve into definite CTDs like systemic lupus erythematosus (SLE), systemic sclerosis, primary Sjögren’s syndrome, mixed connective tissue disease, systemic vasculitis, polymyositis, and rheumatoid arthritis [6].

UCTD is the most common rheumatic disease diagnosed during the first trimester of pregnancy and plays a potential causal role of autoimmune disorders in the occurrence of pregnancy complications [7]. Antinuclear antibodies and inflammation in UCTD may impair platelet activity, unbalance coagulation/anticoagulation, increase the risk of thrombosis, and cause collagen synthesis defect and endothelial dysfunction [8–10]. Therefore, it could increase uterine artery resistance, inhibit placenta development and remodeling, and thus increase the risk of preeclampsia, intrauterine growth retardation (IUGR), small for gestational age infant (SGA), and recurrent spontaneous abortion (RSA) [3, 11–14]. A multicenter retrospective cohort study showed that 21% of pregnancy resulted in miscarriages or stillbirths [15]. Spinillo found that women with UCTD had a higher rate (39.0%) of at least 1 spontaneous pregnancy loss than healthy controls with an odds ratio of 5.92 (95% CI, 2.1–17.8) [14]. Other studies reported that the prevalence of adverse pregnancy outcomes ranged from 27 to 30% in women with UCTD [13, 16].

In addition to adverse pregnancy outcomes, women with UCTD may experience a disease flare and even evolve into definite CTDs during the course of pregnancy and puerperium [17, 18]. The incidence of disease flare was about 24~32%, higher than the 11% in the non-pregnant patients [13, 16]. An average of 30% of UCTD women eventually develop a well-defined CTD [18–21], especially during pregnancy, and require treatments with steroids and immunosuppressants like hydroxychloroquine (HCQ).

There is no consensus or guideline about treatments for recurrent spontaneous abortion (RSA) in pregnant women with UCTD. UCTD shares similar pathogenesis of placental vascular thrombosis as systemic lupus erythematosus +/− antiphospholipid antibody syndrome (APS) for which low-dose aspirin and low molecular weight heparin (LMWH) are used to prevent fetal losses and improve pregnancy outcomes [22, 23]. Therefore, antiaggregation and anticoagulation were considered as a safe way of reducing the risk of miscarriages among women with UCTD [24, 25]. Despite low-dose aspirin and LMWH treatments, women who suffered from RSA with UCTD still suffered from pregnancy morbidity [24, 26].

Taking placental inflammation and imbalance of the immune system of UCTD into consideration, immunosuppression is hypothesized to be a potential rescue. Immunosuppression may also be beneficial in reducing disease recurrence and progression. Currently, commonly used immunosuppressant includes low-dose corticosteroids, HCQ, azathioprine, cyclosporin, and immunoglobulin [3]. In a guideline on prescribing drugs in pregnancy and breastfeeding, corticosteroids and HCQ are compatible with each trimester of pregnancy [level of evidence 1++, grade of recommendation A, strength of agreement 100%], and HCQ should be continued during pregnancy in women planning a pregnancy with rheumatic disease in need of treatment [27]. Corticosteroids and HCQ were used in nearly half of UCTD patients and considered as symptomatic treatment during the non-pregnancy period. But the treatment effect on pregnant women with UCTD was poorly studied and barely acknowledged, and most studies are small-scale observational studies. The studies of treatment in recurrent miscarriage, SLE, and APS showed that prednisone and HCQ could reduce the incidence of disease flares and obstetric complications. A low dose of corticosteroids was proved to inhibit T cells and natural killer (NK) cells, to improve the fertilization rate [28, 29], and to decelerate the progression into rheumatic diseases. Several studies showed that prednisolone of 5–20 mg per day can improve pregnancy outcomes [26, 30–33]. However, there still lacks a large clinical trial to identify optimal treatment. It was observed that HCQ was associated with delayed SLE onset in CTD [34], decrease the risk of disease flare in pregnant SLE patients [35–37], increase the live birth rate, reduce preterm delivery in antiphospholipid antibodies (aPL) positive patients [38], and prevent congenital heart block in neonates exposed to anti-SSA and anti-SSB antibodies [39]. It remains unknown whether HCQ could affect pregnancy outcomes of UCTD women. A study of 133 pregnancies of CTDs showed evidence for the safety of HCQ therapy during pregnancy but no differences in live birth rate [40]. A systemic review showed that HCQ does not appear to be associated with any increased risk of congenital defects, spontaneous abortions, fetal death, prematurity, or decreased numbers of live births in patients with autoimmune diseases [41]. Recently, data from 66 UCTD patients with RSA treated by hydroxychloroquine plus low-dose prednisone combined with anticoagulation in our center seemed promising. There were 66 successful pregnancies (the live birth rate 97.1%) and only 5 obstetric complications in 68 pregnancies. The impact on pregnancy outcome of UCTD is probably less severe than well-established rheumatic diseases; however, UCTD needs to be paid more attention to because of its high prevalence.

Given that RSA in UCTD patients is mediated by autoimmune factors and may be improved by the addition of immune suppressants to anticoagulation. The objective of the ILIFE trial is to assess the efficacy of low-dose corticosteroids and HCQ combined with anticoagulation in treating UCTD patients with recurrent pregnancy loss.

## Methods/design

### Study design

The ILIFE trial is a three-arm, multicenter, open-label, randomized controlled trial (RCT) to assess the efficacy of low-dose corticosteroids and HCQ combined with anticoagulation in treating UCTD women with recurrent pregnancy loss.

**Fig. 1:**
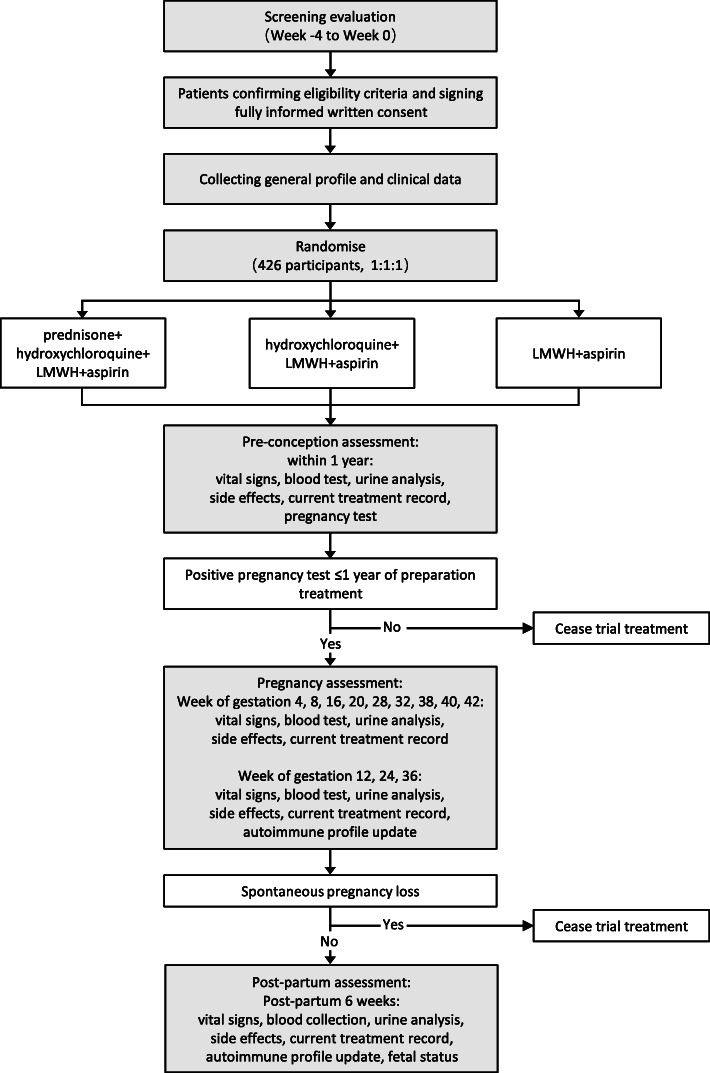
Trial design schema

Patients will be enrolled at rheumatology outpatient clinics in six university-affiliated, tertiary hospitals in China, including Shanghai Renji Hospital, the First Affiliated Hospital of Anhui Medical University, China-Japan Union Hospital of Jilin University, the Affiliated Wuxi No.2 People’s Hospital of Nanjing Medical University, Jiangsu Province Hospital, and Xiangya Hospital of Central South University. The study design is presented in the flowchart in Fig. 1. Standard Protocol Items: Recommendations for Interventional Trials (SPIRIT) are provided as Additional file 1. This study was approved by institutional review boards (IRB) of study sites.

### Recruitment

The study population consists of UCTD women who had experienced at least two miscarriages and are trying to conceive. Rheumatologists in each study site will check inclusion and exclusion criteria of UCTD women. A research assistant will present the study information during recruitment. Eligible patients who provided informed consents will be enrolled in the trial.

The anticipated duration of recruitment is 2 years, and the duration of participation of each patient is about 2 years.

### Inclusion criteria

Women who meet the following inclusion criteria will be eligible to participate in the study:

- At reproductive age (20–40 years old).
- Trying to conceive.
- Diagnosed with UCTD [2]: at least one symptoms or signs suggesting CTD and with at least one presence of auto-antibodies, including antinuclear antibody (ANA), anti-extractable nuclear antigen (ENA) antibodies, anti-double-stranded DNA (ds-DNA) antibody, aPL, and anti-cyclic citrullinated peptide (CCP) antibody, while not fulfilling any classification criteria of a defined CTD.
- Diagnosed with RSA [42]: two or more failed pregnancies of unknown origin in all trimesters.
- Providing written informed consent.

### Exclusion criteria

Women who meet any of the following criteria will be excluded from the study:

- Any known etiology of previous pregnancy loss:

1. Diagnosis of antiphospholipid antibody syndrome.
2. Known paternal, maternal, or embryo chromosome abnormality.
3. Maternal endocrine dysfunction: corpus luteal insufficiency, polycystic ovarian syndrome, premature ovarian failure (follicle-stimulating hormone, FSH ≥ 20 U/L in follicular phase), hyperprolactinemia, thyroid disease, diabetes mellitus, and other hypothalamic–pituitary–adrenal axis abnormality.
4. Maternal anatomical abnormality: uterine malformation (using Thessaloniki ESHRE/ESGE consensus criteria, gynecological examination, ultrasound results, and opinions of obstetricians and gynecologists), Asherman syndrome, cervical incompetence, and uterine fibrosis more than 5 cm (including submucosal and/or intramural).
5. Vaginal infection.

- Any known severe cardiac, hepatic, renal, hematological, or endocrinal diseases:

1. Alanine transaminase (ALT) or aspartate transaminase (AST) more than twice the upper limit of normal.
2. Clearance of creatinine less than 30 mL/min.
3. Leucocytes less than 2.5 × 10^9^/L, hemoglobin less than 85 g/L, or platelet less than 50~10^9^/L.

- Any active infection:

1. Active viral hepatitis including hepatitis B virus (HBV), hepatitis C virus (HCV).
2. Active infection including varicella-zoster virus (VZV), human immunodeficiency virus (HIV), syphilis, or tuberculosis.

- Allergic to prednisone, hydroxychloroquine, LMWH, or aspirin.
- Disease history as follows:

1. Past history of digestive ulcers or upper gastrointestinal hemorrhage.
2. Past history of malignancy.
3. Past history of epilepsia or psychotic disorders.

- Women using assisted conception.
- Women unable to consent.

### Randomization and blinding

The primary objective of this trial is to investigate the superiority of low-dose corticosteroids and HCQ combined with anticoagulation compared with anti-coagulant only in UCTD women with RSA. A third arm of using hydroxychloroquine combined with anticoagulant for a secondary comparison only. It is an open-label trial, but data analysts will be blinded. After obtaining the written informed consent, eligible patients will be randomized to one of the three treatment arms with a ratio of 1:1:1 stratified by site. Randomized numbers will be generated through SAS software at the trial methods center located in Shanghai Renji Hospital. A specially designed sealed opaque two-couplet random allocation envelope will be used for the randomization and allocation. The first couplet is to collect the enroll information and the second couplet is to show the allocation information. The content of the first couplet can be completely copied to the second couplet. Each time a new patient is enrolled in the study, a new envelope will be opened in order. Once the envelope is opened, it cannot be resealed.

### Treatments

Included patients are randomized to three treatment groups: (1) low-dose prednisone (10 mg once daily orally), HCQ (the maximal tolerance dose ranges from 100 to 400 mg daily orally), and anticoagulants using low-dose aspirin (50 mg once daily orally) and subcutaneously administered LMWH once daily with preventive dose (enoxaparin 40 mg or dalteparin 5000 IU or nadroparin calcium 4100 U); (2) HCQ (the maximal tolerance dose ranges from 100 to 400 mg daily orally), and the same anticoagulant treatments as in the first group; and (3) the anticoagulant treatments only. Treatments are initiated before conception (recommended 3–6 months) and stopped at 6 weeks post-gestation or miscarriage or after 1 year of treatment in the absence of pregnancy. Supplements such as folic acid, calcium tablet, and vitamins are allowed throughout pregnancy.

### Assessments and data collection

**Fig. 2:**
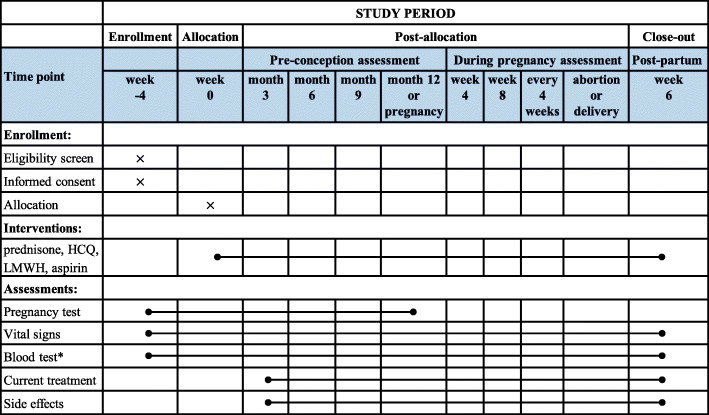
SPIRIT figure: time schedule of enrollment, interventions, and assessments. An asterisk denotes blood count, biochemistry, autoimmune profile, and coagulation profile

The assessments will be conducted at the screening and preparation phases and then routinely throughout the trial. The screening phase will last for 4 weeks. General profiles including age, height, weight, ethnicity, occupation, obstetric and rheumatology histories, medication uses, allergy, and antecedents will be recorded during the screening phase. Following the screening phase is the 1-year pre-conception phase. If participants are not pregnant within 1 year, the treatment and their participation will be terminated. Once pregnancy is confirmed, the pregnancy assessments will start and the index time is set to week 0. Pregnant women will be followed once every 4 weeks in the next 42 weeks gestation period. The last visit will be conducted at 6 weeks after delivery. The assessment will be terminated when spontaneous pregnancy loss or childbirth takes place. Children’s observations will last for 1 year after the end of the study, including vision, hearing, and growth parameters. The schedules for assessments and data collection are showed in Fig. 2. Data will be collected in paper case report forms (CRFs) stored at a secure place and double entered into the electronic trial database.

### Outcome measures

#### Primary outcome

The primary outcome is the rate of live births after at least 26 completed weeks of gestation.

#### Secondary outcomes

The secondary outcomes include adverse pregnancy outcomes and progression of UCTDs:

- The rate of miscarriage (before 24 weeks).
- Premature birth (live birth between 28 and 37 weeks of gestations).
- Concerning the child: intrauterine growth retardation, gestational age and weight at birth, survival at 28 days, safety data at 42 days of life, and congenital abnormality (congenital heart conduction block, neonatal lupus, or malformation).
- The rate of stillbirth (intrauterine death at ≥ 24 weeks).
- Concerning the mother: eclampsia (new-onset hypertension after 20 weeks of gestation, with or without proteinuria more than 300 mg/24 h; obstetric bleeding (antepartum hemorrhage, postpartum hemorrhage); and placental abnormalities (placenta praevia), with or without any organ damage with seizures), infection (infections of the upper and lower respiratory tract, gestational source, urinary tract, and skin), and gestational diabetes mellitus (diabetes diagnosed in the second or third trimester of pregnancy).
- Concerning the UCTD: UCTD flare (the presence of new clinical symptoms like arthritis, rash, Reynolds phenomenon, proteinuria with or without serological activity, which requires a new treatment or an increase of current dose or even hospitalization due to disease activity), UCTD evolution (evolution to SLE, APS, Sjogren’s syndrome, systemic sclerosis, and other defined CTD diagnosed by classification criteria of American College of Rheumatology).
- The rate of conception.

### Sample size

There is very limited evidence on the spontaneous pregnancy loss in pregnancies of women with UCTD and the reported risk rates for RSA varied substantially across studies. The live birth rate of a randomized trial enrolling 364 women with a history of unexplained recurrent miscarriage was 69.1% in the group receiving aspirin plus nadroparin [43]. In another cohort study of women with positive aPL, HCQ can improve the live birth rate from 57 to 67% [38]. A double-blind placebo randomized control trial involving 160 patients with unexplained recurrent miscarriage showed that the addition of prednisolone to heparin and aspirin might be beneficial, with 70.3% of women in the prednisolone group having successful pregnancy outcome versus 9.2% in the placebo group [30]. Data from 68 pregnancies in our department showed that the live birth rate was 97.1% in UCTD women with RSA treated by hydroxychloroquine plus low-dose prednisone combined with anticoagulation.

Our primary comparison is between the combined use of prednisone, HCQ, and anticoagulant with the anticoagulant only. Based on our pilot and limited published data, we hypothesize that the proportion of live birth rates with the treatment strategy using prednisone, HCQ, aspirin, and LMWH is 85% vs 70% for anticoagulant only. We need to enroll 363 women (121 in each group and for the third arm as well) to allow us to detect the difference in spontaneous pregnancy loss between the treatment groups with a power of 0.8 at a two-sided *P* value of 0.05. Since not all women would get pregnant during the trial, we planned to include about 426 participants (142 in each group) on the assumption of 90% of the pregnant rate within 1 year and 5% loss to follow-up [44].

### Data analysis

The primary analysis is to compare the rate of live births after at least 26 completed weeks of gestation between the combined use of prednisone, HCQ, and anticoagulant with the anticoagulant only. All data will be analyzed according to the intention-to-treat approach in which all randomized patients are included and supplemented by per-protocol analysis. For categorical data, frequencies and percentages will be presented. Continuous data will be presented as the mean and standard deviation or median and interquartile range. Comparison between groups will be performed using the independent sample *t* test, Pearson’s *χ*^2^ test, or Fisher’s exact test as appropriate. The primary outcome will be summarized as the proportion of live birth in each group. The absolute differences and relative risks with 95% confidence intervals will be described for the primary and secondary outcomes. The relative risks will be adjusted for age and number of previous miscarriages.

The trial has a single primary endpoint, i.e. rate of live births after at least 26 completed weeks of gestation between the combined use of prednisone, HCQ and anticoagulant with the anticoagulant only. All secondary comparisons, including comparisons to the third treatment arm (HCQ and anticoagulant) will be performed to further inform the primary endpoint and will be interpreted on this basis.

A sensitivity analysis using multiple imputations will assess the robustness of the results to variation in the missing data assumptions. Subgroup analysis will be conducted according to the number of previous miscarriages (2 or ≥ 3). Two-tailed significance tests will be performed at the 5% significance level, without adjustment for multiple testing.

### Trial management

A steering committee comprised of a local principal investigator at each participating hospital will manage the trial. Screening and recruitment will be reviewed regularly at steering committee meetings; protocol deviations (e.g., late administration) will be distinguished from protocol violations (e.g., missed doses). Enrollment or adherence barriers will be addressed by implementing improvement strategies. Relevant clinical and laboratory data submitted to the steering committee will be checked for completeness and accuracy. All investigators will undertake a standardized training for study procedures, data collection, and adverse event reporting. The plans to promote participant retention and complete follow-up include giving a specific follow-up schedule and obtaining at least two phone contacts to ensure communication. The research assistant will contact participants by telephone regularly to improve adherence to medication and assessments. We will record and analyze reasons for nonadherence or nonretention in the study. To maintain confidentiality, CRFs and other records will be identified by a coded number and initials only. Data tools and instruments are stored securely, and access is restricted to authorized study team members.

### Safety monitoring

An independent data and safety monitoring board (DSMB) will meet regularly to ensure patient safety and data quality. Interim analyses of principal safety and effectiveness outcomes will be performed on behalf of DSMB by the trial statistician who is blind to the treatment assignments. Vital signs, which include blood pressure, heart rate, temperature, and respiration rate, adverse events will be monitored regularly throughout the trial. Safety monitoring and surveillance will be collected, processed, reviewed, evaluated, reported, and discussed, including clinical pregnancy outcomes, identified risks of drugs (Additional file 2), laboratory abnormalities, and imaging changes. If serious adverse outcomes or rare possible risks occur, a multidisciplinary safety management team (SMT) will be established in each participating hospital, and the results will be communicated with DSMB. After assessing severity and causality, rheumatologists are responsible for adjusting further treatment plans accordingly, and adverse events will be communicated with DSMB as soon as possible.

### Dissemination

The research team will make the data and the full study protocol available to the public. The findings of the study will be disseminated in academic journals and conference presentations.

## Discussion

UCTD is the most common systemic rheumatic disorder that occurred mainly in women at the reproductive age and is associated with a higher rate of recurrent spontaneous abortion [14]. To date, there has not yet been an optimal therapy for treating RSA of UCTD patients. Among all the possible causes of RSA in UCTD patients, immunological dysfunction is one of the reversible backgrounds affecting pregnancy outcomes. It is recommended that pregnancy in patients with SLE or APS be treated using immunotherapy and anticoagulation therapy. It is known that the immune system imbalance and impaired vascular function in UCTD could contribute to the pathogenesis of preeclampsia and placental vascular thrombosis as is the case in SLE or APS [9, 45]. Therefore, immunotherapy may play a role in reducing the immune system dysfunction and vascular injury caused by UCTD, and then decreasing the adverse pregnancy outcomes. Meanwhile, immunosuppressant may prevent patients from disease recurrence and progression into defined connective tissue disease.

The treatment experiences of pregnant women mostly came from the treatment of recurrent miscarriage, SLE, and APS. The studies of treatment effect on UCTD pregnant women were with small scale instead of randomized controlled study. Based on limited published data, immunotherapy such as low-dose corticosteroids and HCQ showed clinical benefit for RSA in patients with UCTD. There still lacks a large scale, well-designed clinical trial to assess the efficacy of immunotherapy in patients with RSA and UCTD. The ILIFE study is the first RCT to evaluate the efficacy of prednisone and HCQ in this patient population. This trial focuses on ameliorating the pregnancy outcomes, investigating the role of rheumatic diseases in pregnancy, and also exploring how pregnancy modifies the progress of UCTD. We expect that the ILIFE study will provide high-quality evidence to inform treatment decision-making for UCTD women with RSA.

## Trial status

Patient recruitment will begin from August 2019 to August 2021. The trial will be completed by August 2023.

Protocol date and version: June 10, 2019; version 1.1.

## Supplementary information

**Additional file 1.** SPIRIT 2013 checklist: recommended items to address in a clinical trial protocol and related documents.

**Additional file 2.** Adverse events of medications.

## Data Availability

The datasets used and/or analyzed during the current study are available from the corresponding author on reasonable request.

## Abbreviations

ALT: Aspartate transaminase
ANA: Antinuclear antibody
aPL: Antiphospholipid antibodies
APS: Antiphospholipid antibody syndrome
AST: Alanine transaminase
CCP: Cyclic citrullinated peptide
CRFs: Case report forms
CTDs: Connective tissue diseases
ds-DNA: Double-stranded DNA
DSMB: Data and safety monitoring board
ENA: Extractable nuclear antigen
FSH: Follicle-stimulating hormone
HBV: Hepatitis B virus
HCQ: Hydroxychloroquine
HCV: Hepatitis C virus
HIV: Human immunodeficiency virus
ILIFE: The Immunosuppressant for LIving FEtuses
IRB: Institutional review boards
IUGR: Intrauterine growth retardation
LMWH: Low molecular weight heparin
NK: Natural killer
RSA: Recurrent spontaneous abortion
RCT: Randomized controlled trial
SGA: Small for gestational age infant
SLE: Systemic lupus erythematosus
SMT: Safety management team
SPIRIT: Standard Protocol Items: Recommendations for Interventional Trials
UCTD: Connective tissue diseases
VZV: Varicella-zoster virus
