# Supplementary material for: A three-arm, multicenter, open-label randomized controlled trial of hydroxychloroquine and low-dose prednisone to treat recurrent pregnancy loss in women with undifferentiated connective tissue diseases: protocol for the Immunosuppressant regimens for LIving FEtuses (ILIFE) trial
Source: Trials. 2020 Sep 9;21:771. doi: 10.1186/s13063-020-04716-1 (PMC7488113; doi:10.1186/s13063-020-04716-1)
Supplement: Supplementary file 2 — Additional file 2. Adverse events of medications. [file 13063_2020_4716_MOESM2_ESM.docx]

**Additional file 2**

**Adverse events of medications**

| Medications | Adverse effects |
| --- | --- |
| Prednisone | Teratogenicity with slight increased risk of cleft lip  Reduced placental and birth weight  Adrenal cortex suppression  US FDA pregnancy category C |
| Hydroxychloroquine | High doses and prolonged durations associated with central nervous system damage and otoxicity, retinal hemorrhages and abnormal retinal pigmentation  US FDA pregnancy category C |
| Aspirin | First trimester: increased risk of miscarriage, cardiac malformation, and gastroschisis  Third trimester: premature closure of fetal ductus arterious, oligohydramnios, fetal renal impairment, pulmonary hypertension, and prolongation of bleeding time, while no control data in human pregnancy  US FDA pregnancy category C or D (large doses) |
| Low-molecular-weight-heparin | Hemorrhagic events and neonatal hemorrhage  US FDA pregnancy category C |
